# Supplementary material for: Evaluation of the Abbott BinaxNOW COVID-19 Test Ag Card for rapid detection of SARS-CoV-2 infection by a local public health district with a rural population
Source: PLoS One. 2021 Dec 2;16(12):e0260862. doi: 10.1371/journal.pone.0260862 (PMC8638850; doi:10.1371/journal.pone.0260862)
Supplement: S1 Table — (DOCX) [file pone.0260862.s001.docx]

S1 Table

Characteristics of study participants and SARS-CoV-2 detection method and result

|  |  | Total Participants  N (%) | Pos. BinaxNOW | Pos.  RT-PCR | False Neg.^1^ | True Neg.^1^ |
| --- | --- | --- | --- | --- | --- | --- |
|  | Total Participants | 214 | 25 | 37 | 12 | 177 |
| Sex | Female | 142 (66.4) | 14 | 20 | 6 | 122 |
|  | Male | 72 (33.6) | 11 | 17 | 6 | 55 |
| Age | Minor (<18) | 48 (22.4) | 4 | 7 | 3 | 41 |
|  | Adult (18+) | 166 (77.6) | 21 | 30 | 9 | 136 |
|  | 18-29 | 36 (16.8) | 5 | 6 | 1 | 30 |
|  | 30-39 | 40 (18.7) | 6 | 9 | 3 | 31 |
|  | 40-49 | 25 (11.7) | 1 | 3 | 2 | 22 |
|  | 50-59 | 21 (9.8) | 6 | 8 | 2 | 13 |
|  | 60-69 | 18 (8.4) | 3 | 4 | 1 | 14 |
|  | 70-79 | 11 (5.1) | 0 | 0 | 0 | 11 |
|  | 80+ | 15 (7.0) | 0 | 0 | 0 | 15 |
| Race | Asian | 3 (1.4) | 1 | 1 | 0 | 2 |
|  | Black/African American | 1 (0.5) | 0 | 0 | 0 | 1 |
|  | Native American | 6 (2.8) | 1 | 1 | 0 | 5 |
|  | Native Hawaiian/Pacific Islander | 1 (0.5) | 0 | 0 | 0 | 1 |
|  | White | 141 (65.8) | 19 | 31 | 1 | 110 |
|  | Undisclosed | 62 (29.0) | 4 | 4 | 0 | 58 |
| Ethnicity | Hispanic/Latino | 42 (19.6) | 7 | 12 | 5 | 30 |
|  | Not Hispanic/Latino | 113 (52.8) | 17 | 24 | 7 | 89 |
|  | Undisclosed | 59 (27.6) | 1 | 1 | 0 | 58 |
| Symptoms | One or more | 177 (82.7) | 23 | 33 | 10 | 144 |
|  | None | 37 (17.3) | 2 | 4 | 2 | 33 |
| Days Since Symptom Onset | Median (IQR) | 2 (1-3) | 3 (2-3) | 3 (1-3) | 2.5 (1-4) | 2 (1-3) |
|  | 0 – 3 days | 134 (62.6) | 19 | 25 | 9 | 109 |
|  | 4 – 7 days | 39 (18.2) | 4 | 7 | 3 | 32 |
|  | 8 – 10 days | 3 (1.4) | 0 | 1 | 1 | 2 |
|  | 11 – 14 days | 0 (0.0) | 0 | 0 | 0 | 0 |
|  | >14 days | 1 (0.5) | 0 | 0 | 0 | 1 |
| Known Exposure | Yes | 77 (36.0) | 12 | 21 | 8 | 56 |
|  | No | 137 (64.0) | 13 | 16 | 4 | 121 |
| COVID-19 Immunization Status | Fully Vaccinated^2^ | 26 (12.1) | 1 | 1 | 0 | 25 |
|  | In Progress | 5 (2.3) | 0 | 0 | 0 | 5 |
|  | None | 183 (85.5) | 24 | 36 | 12 | 147 |

^1^BinaxNOW result compared with RT-PCR result (gold standard); no false positives occurred.

^2^At least two weeks past final dose of a COVID-19 vaccine
