# Supplementary material for: Evaluation of the Abbott BinaxNOW COVID-19 Test Ag Card for rapid detection of SARS-CoV-2 infection by a local public health district with a rural population
Source: PLoS One. 2021 Dec 2;16(12):e0260862. doi: 10.1371/journal.pone.0260862 (PMC8638850; doi:10.1371/journal.pone.0260862)
Supplement: Attachment — Submitted filename: Response to Reviewers.docx [file pone.0260862.s002.docx]

**Response to Reviewers**

*We sincerely thank the reviewers for their time and for their thoughtful feedback. Line numbers referenced by the authors refer to those in the revised manuscript without track-changes.*

Reviewer #1: I have no comments on the submitted manuscript "Evaluation of the Abbott BinaxNOW™ COVID-19 Test Ag Card for rapid detection of SARS-CoV-2 infection by a local public health district with a rural population"

***Author Response:*** *The authors sincerely thank Reviewer #1 for their time.*

Reviewer #2: The study describes the performance of the BinaxNOW Ag Card for the detection of SARS-CoV-2 virus antigen in clinical samples. The manuscript is well-structured and readily understandable, and the authors conclusions that are drawn from the presented data are plausible. The results are discussed from several perspectives and integrated into current knowledge about the BinaxNOW test performance. However, discussing the BinaxNOW test performance with that of other rapid test products would add further value to the manuscript.

***Author Response:*** *Southwest District Health exclusively used and evaluated the Abbott BinaxNOW™ COVID-19 Test Ag Card for detection of SARS-CoV-2 viral antigen, paired with confirmatory RT-PCR testing for all negative test results. Unfortunately, due to distribution of rapid antigen tests through federal programs nationally, the state of Idaho did not receive other SARS-CoV-2 rapid antigen tests during the study period for direct comparison by this study.*

Reviewer #2: In ll270ff the authors state that, due to the high specificity of the BinaxNOW test, positive test results may require confirmation by RT-PCR. I do not understand why, please explain. A positive result of a high specificity test should be ok?

***Author Response:*** *The authors have reviewed your comments and agree that the data presented in this manuscript indicate that confirmatory testing may not be required with a positive BinaxNOW™ COVID-19 Test result.*

**Other points:**
- Due to the diverse test principles in point of care testing, the precise format (lateral flow assay?) would be helpful

***Author Response:*** *The authors agree with your comments, and have included format specification in the Introduction, page 4 line 68-69.*

- Beginning with citation #10, the numbering is flawed. Please correct

***Author Response:*** *Citation numbering has been reviewed and corrected.*

- Tables 2+3: is somewhat misleading, you could consider to choose another term (e.g. or just )

***Author Response:*** *Table 3 has been removed from the revised manuscript, and has been replaced with a brief summary of the contents of Table 3 on page 10 line 203-204.*

- Is the information in Table 3 relevant for the interpretation or discussion of study results?

***Author Response:*** *We agree that these data are not relevant to the interpretation of the study results. However, we do feel that currently circulating lineage may be relevant in future replications or auxiliary research. For this purpose, we are including the data in sentence format on page 10 line 203-204.*

- Tables 4+5: in the text 25 concordant samples are described, in Tables 4+5 only 24 samples are listed

***Author Response:*** *After reviewing the relevant dataset, the authors determined there was a clerical error, resulting in the omission of a Ct value for one concordant specimen’s RT-PCR assay. The relevant data was housed on an internal server at the Idaho Bureau of Laboratories and was able to be included in all related statistical calculations. Validation of all existing data and calculations was conducted to ensure accuracy. The inclusion of the omitted Ct value has minimal impact exploratory calculations and no impact on the significance of the evaluation of Ct values for concordant and discordant results.*

Reviewer #3: This is a very well written paper. I fully appreciate that authors fully document the limitations of both the study and the rapid test. These limitations in no way affect the importance to public health in the presentation of these data to the public.

***Author Response:*** *The authors sincerely thank Reviewer #3 for their time.*
